# Evaluation of Automatic Facial Wrinkle Detection Algorithms

**DOI:** 10.3390/jimaging6040017

**Published:** 2020-04-01

**Authors:** Remah Mutasim Elbashir, Moi Hoon Yap

**Affiliations:** 1 College of Computer Science and Information Technology, Sudan University of Science and Technology, Khartoum 1111, Sudan; 2 Department of Computing and Mathematics, Manchester Metropolitan University, Manchester M1 5GD, UK; M.Yap@mmu.ac.uk

**Keywords:** facial wrinkles, automatic wrinkle detection, FERET dataset, Sudanese dataset, Jaccard Similarity Index

## Abstract

Facial wrinkles (considered to be natural features) appear as people get older. Wrinkle detection is an important aspect of applications that depend on facial skin changes, such as face age estimation and soft biometrics. While existing wrinkle detection algorithms focus on forehead horizontal lines, it is necessary to develop new methods to detect all wrinkles (vertical and horizontal) on whole face. Therefore, we evaluated the performance of wrinkle detection algorithms on the whole face and proposed an enhancement technique to improve the performance. More specifically, we used 45 images of the Face Recognition Technology dataset (FERET) and 25 images of the Sudanese dataset. For ground truth annotations, the selected images were manually annotated by the researcher. The experiments showed that the method with enhancement performed better at detecting facial wrinkles when compared to the state-of-the-art methods. When evaluated on FERET, the average Jaccard similarity indices were 56.17%, 31.69% and 15.87% for the enhancement method, Hybrid Hessian Filter and Gabor Filter, respectively.

## 1. Introduction

Facial wrinkles are considered to be a facial feature, and appear as people get older [1]. The changes of facial wrinkles depend on the nature of skin and muscle contraction [2]. Therefore, the detection of wrinkles plays an important role in applications that depend on facial skin changes, such as face age estimation [3]. Wrinkles are defined as small furrows or creases in the skin caused by age or expressions [3]. The appearance of skin with wrinkles might show deep creases, and sometimes with surrounding curvature [4].

Over recent decades, different approaches have been proposed to detect facial wrinkles (henceforth, wrinkles). Loden et al. [5] are wrinkle detection pioneers. They proposed an approach to detect wrinkles using silicone to produce wrinkles replica. Based on the replica, they measured the properties of wrinkles. This approach was not efficient due to the difficulties in getting the exact wrinkle replicas as the actual skin morphology. With the development of computerized methods, new approaches have been proposed to automate the detection of wrinkles. Ng et al. [6] proposed a novel method to detect and quantify facial wrinkles automatically, which is known as Hybrid Hessian Filter (HHF). It showed better results when compared to the other state-of-the-art methods [7].

Automated wrinkle detection is an important process for some real-world applications. To bridge the gap in existing research, we evaluate the performance of the state-of-the-art wrinkle detection algorithm on the whole face. Furthermore, we propose to use an enhancement technique [8] to improve the detection rate on the whole face. This idea is inspired by Jerman et al. [8], where they used the enhancement technique on vessel detection and produced promising results. The enhancement method detects facial wrinkles in 2D images using second-order derivatives and Gaussian scale-space. Jaccard Similarity Index (JSI) is used to assess the performance in detecting wrinkles against ground truth. To test the robustness of the algorithms, we evaluate it on two datasets, i.e., 45 images from FERET [9] and 25 images from our in-house Sudanese dataset.

The rest of this paper is organized into the following sections: Section 2 outlines the state-of-the-art methods for wrinkle detection. Section 3 describes Jerman et al.’s enhancement method [8]. Section 4 presents the results and discussion. The paper is concluded in Section 5.

## 2. Related Work

In an earlier work, Kown and lobo [10] studied the appearance of facial wrinkles on different face region to distinguish between young and senior adults. In 2011, Choi et al. [3] proposed a representation scheme of the wrinkles and used it to predict the skin age. Their results showed that face age can be estimated automatically from the images.

Furthermore, other researchers attempted to develop an automatic wrinkle detection algorithm, which considered wrinkle detection as a line or ridge detection problem. Cula et al. [7] developed an algorithm for automatic wrinkle detection in 2D images; their method works on forehead furrow wrinkles and is based on orientation estimation and the frequency of elongated spatial features. They tested their method based on information such as depth and length of the wrinkles.

Ng et al. [4,6,11] proposed a few methods to detect wrinkles; their studies are considered to be benchmark algorithms as they reported good results. In 2014, Ng et al. proposed a novel method called Hybrid Hessian Filter (HHF) [6]; it was used to detect forehead wrinkles in 2D images, and the algorithm was based on Hessian matrix and directional gradient. It was used to detect wrinkles by computing Hessian matrix with all image pixels. HHF outperformed state-of-the-art methods with an average JSI of 75.67%. Hessian Line-Tracking (HLT) is another method proposed by Ng et al. [4], which is considered to be an extension of HHF. Its performance was evaluated on forehead wrinkles in 2D images. The algorithm overcame the HHF problem by tracking the wrinkle lines. HHF was the first step in HLT which was used to extract seeds; after that, optimum pixels were determined to use as starting point. Then, line-tracking was used to determine which pixel belonged to the wrinkle line. Finally, they applied post-processing to remove the noise from the detected wrinkles. The algorithm outperformed other state-of-the-art methods, where they compared it with HHF [6] and Frangi’s method [12]. The accuracy reported by the authors was an average JSI of 84.00%. Furthermore, Ng et al. [11] introduced an extended algorithm to detect all facial wrinkles using their previous method (HLT) [4]. This study was intended to investigate the effects of facial wrinkles on face age estimation.

Batool and Chellappa [13] proposed a wrinkle detection algorithm called fast wrinkle detection. They first used Gabor Filter Bank to extract the image features, then they evaluated the performance of their algorithm on forehead wrinkles. Additionally, Batool and Chellappa [14] demonstrated the use of wrinkles as soft biometrics.

Most of the wrinkle detection methods reviewed above focused on detecting horizontal lines in the forehead region. In 2017, Omaima et al. [15] proposed a modified HHF algorithm to detect vertical lines. This algorithm was used as a wrinkle extraction step for their study in investigating the effects of smoking on facial wrinkles. Modified HHF was the first method used to detect vertical facial lines, but it did not validate the state-of-the-art dataset. Furthermore, Yap et al. [16] extended HHF by introducing a multi-scale filter to extract coarse and fine wrinkles for the facial regions. They used this method in their automated facial wrinkles annotator, which was presented to overcome the difficulty of manual annotation. It showed a good result when evaluated against manual annotation using some selected images from their in-house and FERET dataset.

## 3. Methodology

This section describes the dataset preparation, the preprocessing steps and the wrinkle detection algorithms.

### 3.1. Dataset

Two datasets were used in this research: state-of-the-art face dataset (FERET) and a new Sudanese dataset.

#### 3.1.1. FERET Dataset

**Figure 1:**
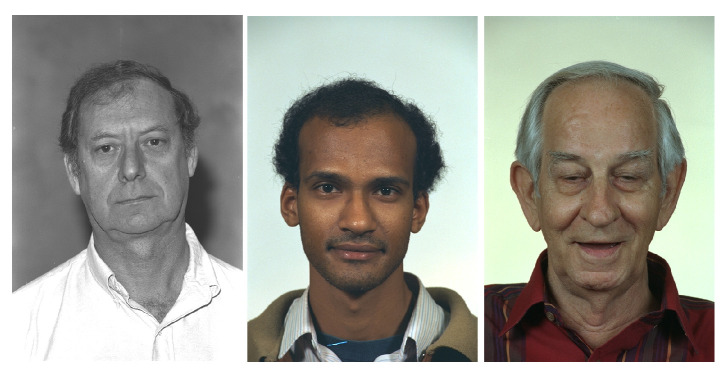
A sample of images from FERET [9].

FERET dataset is one of the benchmark datasets used to assess the studies of facial analysis. Ng et al. used FERET in [6] to assess their wrinkle detection method. The dataset was collected under a controlled environment. It was made available for researchers to test the algorithm of facial recognition [9]. FERET dataset consists of 2366 images collected from 994 subjects, with age range between 10 and 70 years old. When compared to FGNET [17], FERET has better quality as it was collected under a controlled environment and not reproduced by scanning existing photographs. Figure 1 shows some sample images from FERET.

#### 3.1.2. Sudanese Dataset

**Figure 2:**
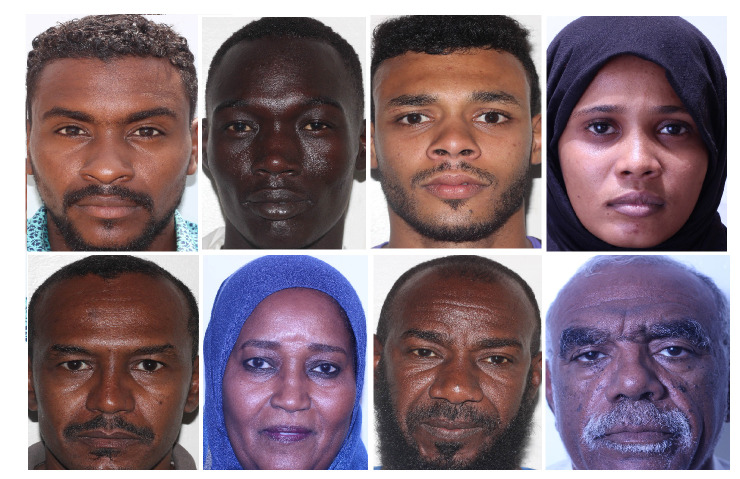
A sample of images from Sudanese dataset.

Sudanese dataset was collected under natural light settings. It was taken in different environments under ambient light. The dataset consisted of 1287 images collected from 136 subjects (130 male and 6 females). The age range is between 18 and 80 years old, inclusively. For each subject, there are 7 to 10 images with different facial expressions and poses. We distributed a questionnaire including personal data and smoking status, alongside a consent form to obtain permission to use participants’ photos for research purposes. The images were taken using Cannon EOS 600D, with flash turned on. Figure 2 illustrates a sample of images from Sudanese dataset.

### 3.2. Face Alignment

**Figure 3:**
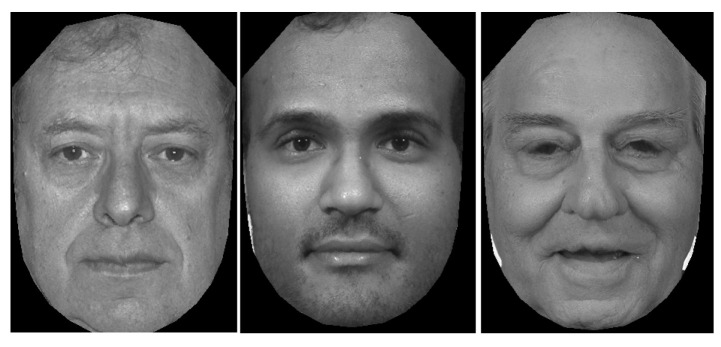
A sample of images from FERET dataset with face alignment.

For face detection, we use a freely available technique—Face++ detector [18]. Face++ face detector uses a deep learning approach to detect the face. There is a total of 88 facial landmarks detected on a face image, with 24 points of them on face contour, and another 64 points inside the face region (mouth, nose, and eyes). For face alignment, a triangulated mesh was obtained using Delaunay’s method [19] on a mean shape. Figure 3 represents the result of face alignment on a sample of FERET images.

### 3.3. Wrinkles Detection

**Figure 4:**
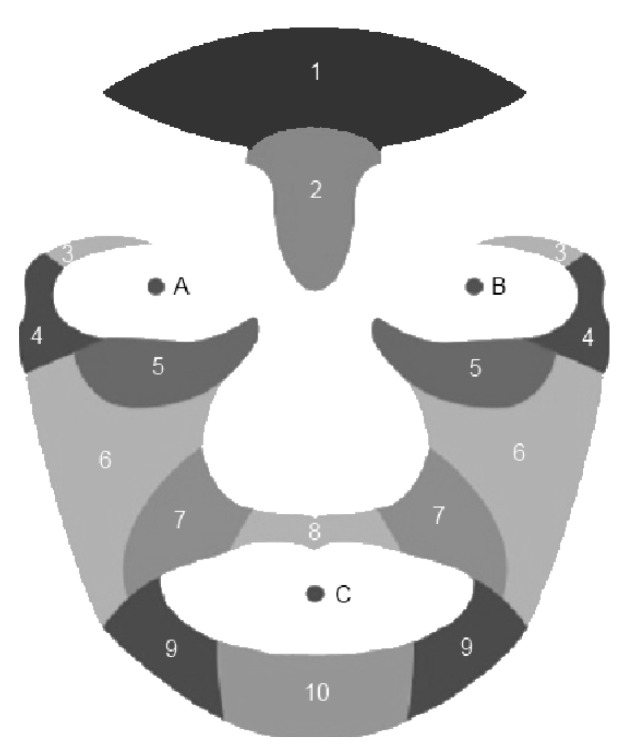
Facial mask with ten regions as defined by [20].

In this step, a face template or mask was used with ten predefined wrinkle regions with fixed coordinates for mouth and eyes [20]. The mask was divided into 10 regions: forehead, glabella, upper eyelids, crow’s feet (or eye corners), lower eyelids (or eyebag), cheeks, nasolabial grooves (or nasolabial folds), upper lips, marionette and lower lips, as illustrated in Figure 4. Each facial image was normalized to the mask by using piecewise affine warping [21]. Finally, these ten regions were used to evaluate the wrinkle detection algorithms.

### 3.4. Proposed Method

Facial wrinkles are considered to be an important feature of aging. Accurate wrinkle detection is a cornerstone in several image-based applications [3,22]. Many wrinkle detection methods were proposed, some of them with good results [4,6]. In this work, we propose use of Jerman et al.’s method [8] for 2D image wrinkle detection (henceforth, enhancement method). Originally, this method was used to detect vessels [8]. It used second-order intensity derivative or Hessian for all image points, in addition to Gaussian scale-space of the image. For details of the method, please refer to Jerman et al. [8]. To illustrate our adoption of the method for wrinkle detection, we summarise the method as follows. Let I(x) denote the intensity of image at coordinate *x*, the Hessian matrix of I(x) at scale *s* can be represented by:

(1) $$H_{i,j}=s^{2}I\left(x\right)*G\left(x,s\right)$$

where i,j=1,…,D, $x={\left[x_{1},\ldots ,x_{D}\right]}^{T}$, and G(x,s) is Gaussian method, which is calculated by:

(2) $$G\left(x,s\right)={\left(2\pi s^{2}\right)}^{\frac{-D}{2}}exp\left(\frac{-x^{T}x}{2s^{2}}\right)$$

To determine the wrinkle shape and direction, eigenvalues ($\lambda_{1},\lambda_{2}$) of Hessian matrix have been used. According to [8], the relationship between two eigenvalues can be represented as:

(3) $$|\lambda_{2}\left|\gg \right|\lambda_{1}|$$

The sign of eigenvalues indicates a bright (dark) structure on dark (bright) background, so it can be represented as in Equation (4):

(4) $$\begin{array}{c} \lambda_{i}:=\{\begin{array}{c} -\lambda_{i}:brightstructureondarkbackground\\ \lambda_{i}:darkstructureonbrightbackground \end{array} \end{array}$$

The vessel detection process was based on the brightness/darkness of vessel structure compared to the background. We can use a similar concept in wrinkle detection. Jerman’s et al. [8] enhancement method was used to detect a vessel in 2D and 3D images. It detected all types of vessel structure (elongated, rounded, and elliptic structures), which were not found in other vessel detection methods. Therefore, we used Jerman et al.’s method [8] to investigate how it would perform on wrinkle detection.

To enhance the algorithm, the measures of structural isotropy and anisotropy of diffusion tensors (ratio of eigenvalues) which were used by Peeters et al. [23] can be applied to Hessian matrices as proposed by Jerman et al. [8], this can be illustrated in

(5) $$V=|\lambda_{1}\lambda_{2}\lambda_{3}|{\left[\frac{3}{|\lambda_{1}\left|+\right|\lambda_{2}\left|+\right|\lambda_{3}|}\right]}^{3}$$

Function *V* can be enhanced to indicate elongated structures. The equation is modified by substituting $\lambda_{1}$ by $\left(\lambda_{2}-\lambda_{1}\right)$

(6) $$V=|\left(\lambda_{2}-\lambda_{1}\right)\lambda_{2}\lambda_{3}|{\left[\frac{3}{|2\lambda_{2}-\lambda_{1}\left|+\right|\lambda_{3}|}\right]}^{3}$$

The output from function *V* in Equation (6) had bad responses for low magnitude of $\lambda_{3}$ and $\lambda_{2}$, which was overcome by the calculation of $\lambda_{3}$ at each scale of *s*. Since the enhancement method was intended to detect facial wrinkles in 2D images, $\lambda_{3}$ was substituted by $\lambda_{2}$ to regularize Equation (6) to fit 2D images, so $\lambda_{2}$ is calculated at each scale of *s* as in Equation (7).

(7) $$\begin{array}{c} \lambda_{p}\left(s\right)=\{\begin{array}{c} \lambda_{2}\;:\;if\lambda_{2}\geq Tmax_{x}\lambda_{2}\left(x,s\right)\\ \lambda_{2}\geq Tmax_{x}\lambda_{2}\left(x,s\right)\;:\;if0\leq \lambda_{2}\leq Tmax_{x}\lambda_{2}\left(x,s\right)\\ 0\;:otherwise, \end{array} \end{array}$$

Using the output from Equation (7). Function *V* is modified as represented in Equation (8).

(8) $$V=\lambda_{2}^{2}\lambda_{p}{\left[\frac{3}{2\lambda_{2}+\lambda_{p}}\right]}^{3}$$

In this equation $\lambda_{1}$ has been eliminated to ensure normalized function response [8]. Finally, an area threshold is applied to each image to extract the region of interest (connected pixels), which represents the wrinkles. In this work, we set the value of scale *s* to 0.5, 1.0, 1.5, 2.0 and 2.5. We set the parameter tau(t) to 0.5 and 1.0.

### 3.5. Performance Assessment

To evaluate the performance of the wrinkle detection algorithms, we used Jaccard Similarity Index (JSI) or Jaccard similarity coefficient. Originally, JSI was introduced by Paul Jaccard [24]. In 2015, Ng et al. [4,6] used JSI to evaluate the performance of their wrinkle detection methods. The JSI was calculated by using Equation (9).

(9) $$JSI\left(A,B\right)=\frac{\left|A\cap B\right|}{\left|A\cup B\right|}$$

where *A* and *B* are the two binary masks representing manually annotated wrinkles and predicted wrinkles using computerized method). The result of experiments will be reported in the next subsections.

#### 3.5.1. Experiments Using FERET Dataset

To evaluate the performance of the enhancement method, we compared it with two state-of-the-art methods—HHF [6] and Gabor Filter [13]—by reproducing these methods based on the codes we have obtained from the author of the algorithms. 45 selected images from FERET [9] were used to test the three methods.

More experiments were conducted to show how the enhancement method would perform in detecting the wrinkles in ten facial regions individually.

#### 3.5.2. Experiments Using Sudanese Dataset

To test the robustness of the enhancement method in detecting facial wrinkles, further experiments were conducted using 25 selected images from Sudanese dataset. The experiments were based on the state-of-the-art methods, HHF [6] and Gabor Filter [13] in addition to the enhancement method. The results showed that the wrinkle detection was poor for all methods. When repeated on the state-of-the-art methods, we used the default parameters. For the enhancement method, we conducted empirical study for t=0.5,0.6,…,1.0, the best result was achieved at t=1.

## 4. Results and Discussion

Unlike previous wrinkle detection methods, which were used to detect forehead horizontal wrinkles [4,6,13], we evaluated the performance of enhancement method on the whole face on 45 images selected from FERET dataset [9] and 25 images selected from Sudanese dataset. Each selected image was annotated by the researchers using hand labeling. The output annotated images were used as ground truth to evaluate the performance of enhancement method in detecting facial wrinkles.

**Figure 5:**
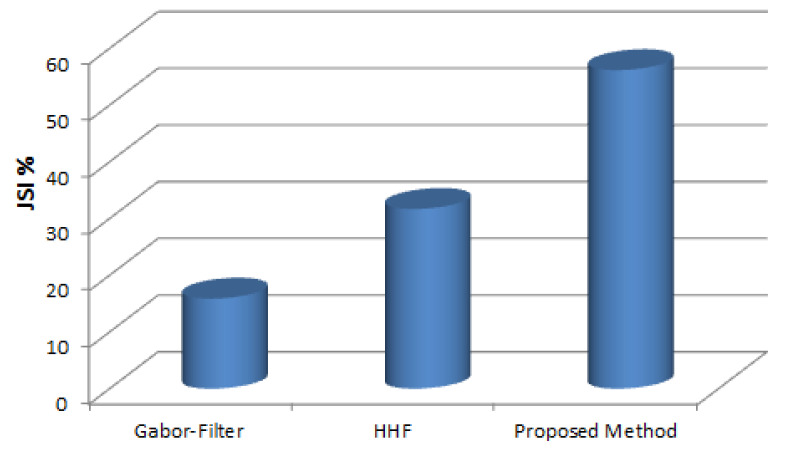
The accuracy of automatic wrinkles detection in JSI.

Figure 5 illustrates the experimental results, where the enhancement method performed better than HHF [6] and Gabor Filter [13]. It achieved an average JSI of 56.17%, where HHF is the second best, with an average JSI of 31.69%, and Gabor Filter achieved an average JSI of 15.87%.

**Figure 6:**
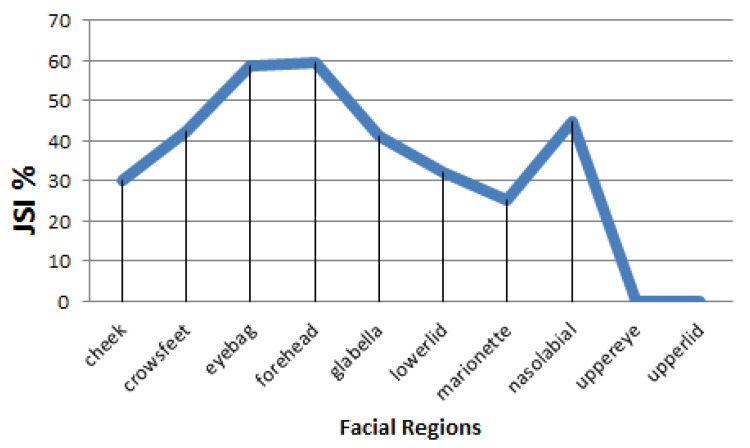
Average JSI of automatic wrinkle detection for ten face regions.

More experiments for enhancement method were conducted to test the detection of wrinkles for each of the ten face regions taken individually. The result is shown in Figure 6, where the enhancement method performed better in forehead and eyebag areas, while the detection rate in area of upper eye and upper lid is very poor.

**Figure 7:**
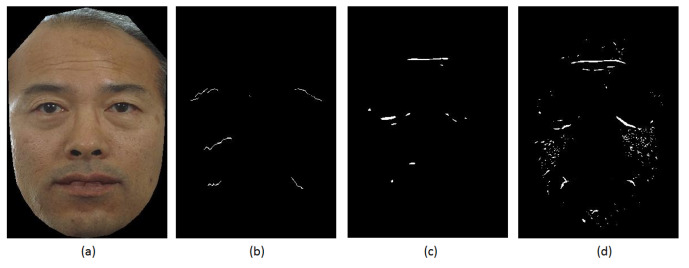
A comparison between the wrinkle detection results of three methods: (**a**) Original image; (**b**) Gabor Filter; (**c**) HHF; and (**d**) Enhancement method.

Although this experiment showed that enhancement method outperformed HHF and Gabor filter in detecting all facial wrinkles, more effort is needed to improve the enhancement algorithm to detect facial wrinkles. The method has some drawbacks: (1) It has a problem with illumination (it generates false positives under different illumination settings); and (2) It generates false detection on hair, such as moustache and cheek. Figure 7 visually compares the result of three methods on an example of FERET image [9].

**Figure 8:**
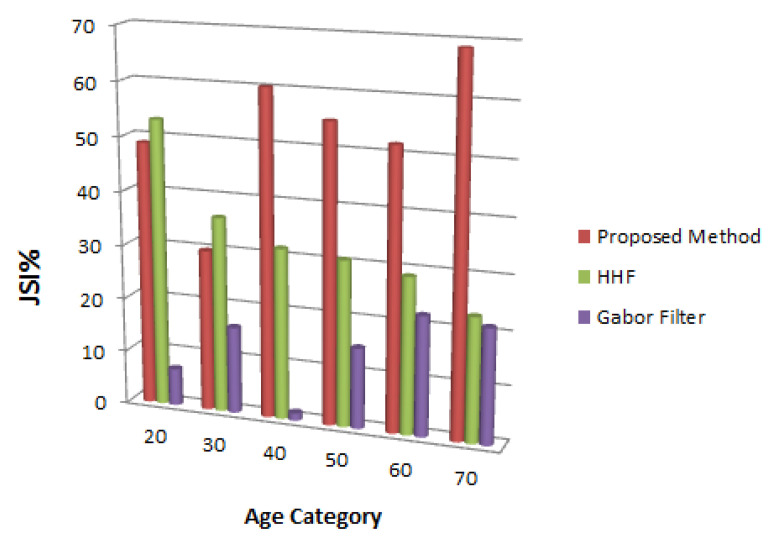
A comparison of the wrinkle detection algorithms on different age group.

Figure 8 shows the output from the experiments according to age group. The figure shows the enhancement method outperforms the other methods in detecting facial wrinkles of middle age and older ages, while HHF produced better results in detecting wrinkles of the younger age group.

In this study, we tested the robustness of wrinkle detection methods on different skin type (mostly brown to dark skin), i.e., on our in-house Sudanese dataset. The evaluation showed poor results for all methods with an average JSI of 27.26%, 9.43%, and 17.6% for HHF, Gabor Filter, and enhancement method, respectively. Although the overall result was poor, HHF showed better results in detecting wrinkles for Sudanese dataset. We can attribute the bad result to the nature of the images, i.e. they were not taken under a controlled environment. In some worst case scenarios, the participants were exposed to direct sunlight. These factors are commonly known in computer vision tasks and affected the result for all the automated wrinkle detection algorithms.

As mentioned in Figure 8, the result of detecting wrinkles in young people’s faces was better when we used HHF method [6]. Additionally, the HHF method has shown better results in detecting wrinkles on the Sudanese dataset. One of the reasons for this is probably associated with the fact that most of the participants’ age in the Sudanese dataset fell between 20 and 40 years old.

Furthermore, for the enhancement method, we have conducted empirical study for t=0.5,0.6,…,1.0; the best result was achieved at t=1 with an average JSI of 20%.

## 5. Conclusions

Existing wrinkle detection methods focus on detecting forehead wrinkles [4,13], owing to their centrality to many applications like age estimation and soft biometric. Many methods focus just on detecting horizontal lines, while it is important to consider vertical lines in all facial regions rather than just forehead wrinkles. This paper proposed to use an enhancement method [8] to detect wrinkles in 2D images using second-order derivatives and Gaussian scale-space of image. We compared the performance of the enhancement method to state-of-the-art methods, i.e., HHF [6] and Gabor Filter [13]. The experiments showed the enhancement method performed better than other state-of-the-art methods on the FERET dataset, but not on the Sudanese dataset. Additionally, we evaluated the performance of the enhancement method in detecting wrinkles on ten face regions. The experiments showed that the enhancement method performed better in the area of the forehead and eyebag, while it gave very low detection rate in upper eye and upper lid areas. Moreover, some experiments were conducted to assess the enhancement method, HHF, and Gabor Filter using selected images from the new Sudanese dataset. The result was poor for all methods. Overall, the enhancement method performed better than other state-of-the-art methods in detecting face wrinkles on a subset of the FERET dataset, but future work is required to improve the algorithm, especially in false wrinkle detection for hair and under different illumination.
